# A Case of Peri-Uterine Cellulitis Terminating in Abscess

**Published:** 1885-09

**Authors:** W. T. Baird

**Affiliations:** Albany, Texas


### A CASE OF PERI-UTERINE CELLULITIS TERMINATING IN ABSCESS.

BY W. T. BAIRD, M. D., ALBANY, TEXAS.

Reported for THE ATLANTA MEDICAL AND SURGICAL JOURNAL.

May 15, 1885, I was called to see Mrs. Emma Ezell, of this place, aged 30, married and the mother of two children, the youngest two and one-half years old. Patient of bilious temperament, tall, with large form. Had always enjoyed good health until after the birth of her first child, now seven years old, since which time she has complained most of the time with some form of pelvic disease, for which she presented herself to my friend, Dr. H. L. Smith, of this place, for treatment on March 1st, last.

He made a caustic application to the os and cervix uteri, which produced considerable pelvic soreness at the time, and he found it neeessary to repeat the application in one week afterwards. Intense pelvic pain immediately followed this application, and she at once took to her bed with "*typhoid fever*," with which she constantly suffered until I saw her and took charge of her case, having in the meantime, three weeks prior to my first visit, an alarming attack of uterine hemorrhage, which Dr. Smith succeeded in arresting at a time when she was considered as at the very point of death. I found her with a temperature of 102; pulse 120; tongue coated with a thick, yellowish fur, tip and edges red, and a red stripe extending down its superior surface; no appetite; bowels constipated; pain constantly complained of in epigastric, hepatic and right inguinal region, and extending down right lower extremity; body extremely emaciated. I found in the hypogastric region, a little to the right of the median line, a tumor, about the size of a large orange, which appeared to have arisen from the pelvis, in the neighborhood of the right ovary. This tumor was hard and exquisitively tender, and I was informed by the family that it had existed there about two weeks, and that it had constantly increased in size since it was first discovered. On digital examination per vaginum I found the cervix small, soft and free from tenderness. The pelvic roof was, throughout its entire extent, tender, dense and inelastic, but the tumor could not be discovered projecting into the vagina, and upon bi-manual examination the tumor seemed to be much nearer the abdominal surface than the vaginal—mictiuration, difficult. Anodynes had been freely administered throughout the course of her disease for the purpose of controlling pain and securing rest, but they had been singularly ineffective, as she had slept but little, and her waking hours were full of pain, and their effect upon the digestive and assimilative functions had been most deleterious. Poultices, as hot as could be borne, had been freely used. I diagnosed the case as one of peri-uterine cellulitis, and conceived the indications to be, to control the pain from which she was suffering, secure rest, reduce her temperature, and improve digestion and assimilation, and thus support her vital powers until suppuration supervened, then to evacuate the pus and institute some means whereby the formation of it could be arrested and the sac containing it be absorbed. As she was heartily tired of internal medication and the external application of poultices, I determined to try the virtue of electricity, especially as I regarded it as a remedy which would meet and fulfill more of the indications in her case than any other *one* remedy of which I had any knowledge. 1st. By its stimulating effect upon the various glandular structures of the system, I would be enabled by it to "arouse the secretions," and thereby improve her appetite, digestion and assimilation. 2d. Through its sedative influence I would be enabled to control her pain, secure rest, and reduce her temperature, effects which anodynes and antipyretics had failed to secure. 3d. Through its favorable influence on nutrition I expected to secure its *tonic* effects, and thus, not only support and sustain her rapidly waning vital forces, but to favorably effect those vital changes which are necessary to transform the exuded inflammatory products into pus, the early production and evacuation of which I regarded as elements of safety in her case. I therefore commenced the treatment by the use of a current from the primary in combination with the first and second induction coils of a Jerome Kidder machine, applying the positive pole by labile applications over the full length of the spine, and the negative over the stomach and bowels, concluding the treatment by allowing the positive pole to rest over the lower sacral region, and the negative over the tumor in the hypogastric region, for ten minutes, using a current as strong as she could bear without discomfort. Time occupied by first seance, thirty minutes. The next morning she stated that she had enjoyed the best night's rest which she had been able to procure since her sickness began.

Repeated the electrical treatment and prescribed a saline laxative. For the sake of brevity, I will state that the faradic current was used daily until June 20th, in about the same manner as above set forth, with the exception that, as she improved so that she could be moved with less diffculty, each treatment was concluded by general applications of the positive pole over body and limbs, and as she became accustomed to the electrical stimulus, the length of the seances were gradually extended to one hour. No medicines were used except saline laxatives in sufficient quantities to keep her bowels freely open each day.

Under this treatment her fever rapidly subsided; her pulse became slower, fuller and softer; her tongue presented a clearer aspect—the extreme redness of tip, edges and center was materially modified; her appetite and digestion were improved (without, however, any perceptible gain in the quantity of flesh); her pain entirely subdued, and she was enabled to obtain refreshing rest, both day and night.

The tumor was soon arrested in its growth, and it gradually softened until at this time (June 20th) fluctuation could be distinctly detected in it, and upon the introduction of a hypodermic needle through the abdominal walls, I was able to fill the barrel with yellow, creamy and inodorous pus. As the sac seemed to be nearer the abdominal than the vaginal surface, I determined to evacuate it through the abdominal walls; and therefore, selecting a point one and one-half inches above the pubes, and one inch to the right of the median line, after having her void her urine, I introduced a No. 2 aspirating needle, previously thinly coated with a squibbs solution of gutta-percha to within one-fourth of an inch of its point, well into the abscess and aspirated from it one quart of pus of the character above spoken of. I now injected the sac (by reversing the action of the aspirator) with warm carbolized water, then drew it out; then continued to repeat this operation until I had washed it out *twelve times* and the water returned clear.

I now filled the sac with warm water, impregnated with chloride of sodium, and, removing the aspirating tube from the needle, connected it (the needle) with the negative pole of a twelve cell McIntosh galvanic battery, and, commencing with two cells, closed the circuit by applying the positive pole of the battery by means of a common sponge-holder to the abdominal surface within one inch of the location of the needle. Upon bringing another cell into the circuit she stated that she distinctly felt a "*terrible bubbling*" on the inside in the vicinity of the point of the needle; it, however, gave her no pain. This "bubbling" I of course attributed to the evolution of hydrogen gas, and it was sufficient evidence to me that the current from the battery was accomplishing the purpose for which it was being applied, namely—decomposing the saline matter in the sac, by which process I expected to arrest suppuration on its inner surface and favor its absorption.

I added four more cells to the circuit, making the full number six, and allowed the circuit to remain closed six minutes; then disconnecting the battery from the needle, and reconnecting the aspirator, I removed about one-half of the water from the sac and left the remainder to be absorbed. During this somewhat tedious operation, which consumed three hours of time, my only assistant was my young, though efficient friend, B. F. Church, to whom I take this method of acknowledging my obligations.

After this, her improvement was rapid. In one week she was able to sit up in a chair, and in two weeks to ride in a carriage and visit her friends, and at this writing (July 20th), she is conducting the affairs of her household. The tenderness and soreness at the recent site of the abscess rapidly disappeared, and the walls of the sac seem to have been completely absorbed, and there has been no appearance of any re-accumulation of pus.

The points in this case which I regard of sufficient interest to justify this detailed report are, first, her extremely exhausted condition, evidently due to the presence of the tumor, and it in a condition which plainly showed that she yet had several weeks of suffering in prospect before it could be so disposed of that she would be entirely free from this source of irritation and debility. Second, the means employed to sustain her rapidly waning vital forces. Third, the measures adopted to destroy the suppurating surface of the sac and favor its absorption.

We are repeatedly and emphatically told by Beard and Rockwell, and they present to us numerous instances *to prove*, beyond the liability of a doubt, that electricity is a "*powerful stimulating sedative tonic*;" and yet the profession is slow to adopt and use it as such.

Former experience had taught me the inestimable value of this agent in cases of extreme debility, and I adopted it in this case with a confidence born of conviction, that it would not disappoint my most sanguine expectations.

It may be asked if I do not think it would have been to her advantage to have combined with the electrical treatment some of the ordinary tonic remedies. I believe it would have been, but as her improvement was so satisfactory to herself and friends, at her earnest solicitation I withheld them.

That thirty-five days were consumed in perfecting suppuration in the sac goes far to indicate the exhausted condition of her nerve forces, for we may look upon the formation of pus from inflammatory products as a vital process, and, as such, requiring an expenditure of nervous force to consummate it. I regard that the indications were plain for the use of electricity in her case, *as being the most direct nerve stimulant and tonic* of which we have any knowledge. That it acts as a direct stimulant to the digestive organs, enabling them to perform their functions with more ease and rapidity, and upon the assimilative organs, thereby causing them to appropriate more nutritive material, and thus directly favor the nutrition of the tissues, was well exemplified in this case.

As to its sedative influence, allaying pain and securing rest, I may only mention, as it was evinced from the date of the first application, nor did it fail to exert this influence to a highly satisfactory degree during the time in which it was employed in her case. The method employed to arrest the suppurative process and favor the absorption of the sac, I don't remember to have ever seen recommended by any one else. It may be observed that had I punctured the sac through some part of the vaginal roof, so that drainage could have been secured, there would have been no occasion to have resorted to *any* means for this purpose; that the sac, properly drained, nature would have taken care of it. This is certainly true, but I could find no place on the vaginal surface where I thought I would be justified in making the puncture. It is true, Professor Brickmell, of New Orleans, says that pelvic abscesses "should always be evacuated pervaginum." But, on the other hand, Professor T. G. Thomas tells us that there are cases in which it is best to aspirate through the abdominal walls, and I undoubtedly regard this as belonging to that class of cases. In aspirating pelvic abscesses through the abdominal walls, I conceive the principal objection to be that, no drainage being established, the almost certain liability of a re-accumulation of pus, and this too, frequently, after the injection into the interior of the sac of the tincture of iodine and other stimulating remedies, with a view of preventing it. Having a knowledge of the facility with which secreting surfaces, containing aqueous contents which are impregnated with various salts, can be destroyed by electrolysis, I regard it as plain that the pyogenic membrane of an abscess could likewise be destroyed and absorption of it promoted if it were placed in like condition, which would simply be *to remove all of the pus from its interior, then fill it with water containing some of the chlorides.*

That this theory was well grounded, the sequel in the above case clearly proves, and I have made this report with the hope that others, who have large abscesses to deal with, where drainage and an arrest of the suppurative process and the absorption of the sac become the perplexing problem that give this safe, cyrllea and I hope efficient method a trial.

